# Assessment of the bioaccessibility of zinc in the selected biofortified food grains

**DOI:** 10.1038/s41598-024-67856-3

**Published:** 2025-03-03

**Authors:** Kazi N S Rafi, M G Aziz, Mohammad Amirul Islam, Sarif Istiak Akash, Md. Jakariya, Moupia Rahman

**Affiliations:** 1 Department of Food Technology and Rural Industries, Bangladesh Agricultural University, Mymensingh, 2202 Bangladesh; 2 Department of Agricultural and Applied Statistics, Bangladesh Agricultural University, Mymensingh, 2202 Bangladesh; 3 Department of Environmental Science and Management, North South University, Dhaka, 1229 Bangladesh

**Keywords:** Biofortification, Bioaccessibility, Hydroponics, Atomic absorption spectrophotometer (AAS), Micronutrient, Fractional factorial design analysis, Plant regeneration, Assay systems

## Abstract

Biofortification of zinc (Zn) is a great means of eradicating Zn deficiency, essentially in developing countries. Current study has evaluated the influence of Zn treatment on bioaccessibility of Zn in food grains along with germination assays. Edible (seed) and inedible (root) portions of BARI Gom 28 (*Triticum aestivum*), BARI Chola 5 (*Cicer arietinum*) and BARI Mung 6 (*Vigna radiata*) sprouts were analyzed for Zn bioaccessibility. The highest Zn (44.50 ppm) was extracted from the seed of BARI Chola 5 at 50 ppm Zn and the lowest (0.45 ppm) was extracted from the root of BARI Mung 6 at control treatment. The highest Zn bioaccessibility percentage (90%) was observed at the seed of BARI Chola 5 and the lowest percentage (62%) at the root of BARI Gom 28 sprouts at 25 ppm Zn treatment. After optimizing fractional factorial design analysis, maximum Zn response is observed in the seed of BARI Chola 5 at 50 ppm Zn treatment with 91.06% composite desirability. Germination percentage, fresh and dried weight of sprouts, sprout length, seed vigor and biological yield showed better results at 50 ppm Zn. Thus, biofortification of food grains through hydroponics approach using control, 25 ppm and 50 ppm Zn treatments produced contrasting effects on Zn bioaccessibility.

## Introduction

Zinc deficiency is a well-documented public health concern in most developing countries as this essential micronutrient is one of the most important nutrient for human well-being^1^. The deficiency of Zn causes failure of the immune system, delayed wound healing, physical growth retardation, etc. The main reason behind Zn deficiency is due to very poor dietary diversity and limited intake of dietary Zn^2,3^.Cereals and legumes are staple food of people in developing countries and hence can be one of the key sources to ensure the presence of Zn in their daily diet^4^. The US recommended dietary allowance (RDA) of Zn ranges from 2 to 12 mg/day (2 mg/day for children, 8 mg/day for adult women, 11 mg/day for adult men, pregnant women need about 11mg/day and for lactating women 12 mg/day is essential)^5^.Usually ZnSO_4_, ZnO and Zn-EDTA are used as a source for Zn biofortification. However, most effective Zn source are ZnSO_4_ and ZnO. Seed soaking in ZnSO_4_ and ZnO solutions at higher concentrations reduce phytic acid, an anti-nutrient that limits the bioaccessibility of zinc, thereby improving Zn bioavailability. Also ZnO increases the chlorophyll, total phenols, and antioxidant activities in sprouts, very small size (1–100 nm), increased stability, reactivity and they may have higher absorption efficiency^6^.

Sprout of food grain is the product of germination process that reactivates the seed metabolism and causes the conversion of starch to simple carbohydrate, complex protein to a simple amino acid, complex lipids to simple fatty acid and also increases the bioaccessibility of minerals. Germination also causes the degradation of many anti-nutrients, such as: trypsin inhibitors, phytates, and tannins which interfere the absorption process of nutrients into human body^7^. Optimum temperature for sprout growth is 28.5 °C^8^. However, sprouting behavior in laboratory conditions, using hydroponics give better results compared to field applications^9^.Among several potential target crops, sprouts are considered suitable for mineral biofortification because of their short growth cycle, high content of nutrients, and low level of anti-nutritional factors like phytate^6^.

Various research approaches are ongoing to mitigate hidden hunger problems like Zn deficiency. Such as, increasing dietary diversity, fortification of foods, nutrient supplementation, and crop biofortification. Among these strategies, diversification of diet and biofortification with Zn are considered the most sustainable approach to mitigate Zn deficiency in susceptible populations. Crop biofortificationcan be done through different approaches *i.e.,* genetic biofortification, transgenic approach, agronomic approach and natural genetic variation^10^.Agronomic biofortification has advantages over other approaches because it can be implemented in a wide range of crops and cultivars. Biofortification increases the bioavailability of necessary trace nutrients in the edible parts of a crop plant. In the case of the general fortification process, nutrients are added to food when they are processed but biofortification makes the food more nutritious by developing it in a nutrient-rich environment. Therefore, it could help in addressing malnutrition issues under different circumstances relatively quickly and despite the enriched nutrition, the bio-fortified crops also show resistance to insects, drought and higher yield^11^.Usually agronomic biofortification utilizes a hydroponic approach to seeds prior to planting which includes direct soaking or germination of targeted crop seeds in a nutrient enrich solution^6^.

Bioaccessibility provides valuable information to select the appropriate dose and food matrix to know about the nutritional efficacy of food^12^. Bioaccessibility can also be defined as the fraction of nutrients that become available for absorption after being released from the food matrix in the GI tract^13^.The amount of bioaccessible Zn content in treated crops depends on the type of Zn source used and their amount of application. It is scientifically proven that higher amount of ZnO application will result in higher amount of Zn bioaccessibility^6^.

In Bangladesh, the national status of Zn deficiency is still at an alarming condition. The overall zinc deficiency among children is 31% having a prevalence of about 10% higher in rural children in comparison to urban^14^. Also mass people of Bangladesh cannot get enough Zn rich animal food because of the low income issue^15^. In this circumstance, current investigation is undertaken to develop Zn biofortified, sprouted grain and a low cost vegetative source of food which can play an important role in eradicating Zn deficiency and establish nutritional security for the people of all ages in Bangladesh.

In this study, all seeds were germinated and sprouting behavior was observed in laboratory conditions by hydroponics approach using zinc oxide solutions as priming media for better germination performance, biological yield, sprout growth and better enrichment of BARI Gom 28 (BW 28), BARI Chola 5 (BC 5) and BARI Mung 6 (BM 6) grains. Keeping the observations above in mind, this investigation has been undertaken for the following objectives.

(a) To assess a few germination assays of BW 28, BC 5 and BM 6 food grains by hydroponics approach using different levels of Zn treatment.

(b) To profile the level and bioaccessible percentage of Zn in the edible portion (seed) and inedible portion (root) of the germinated sprouts after biofortification.

## Results

### Physiological assay of grains

Screening the best quality food grain seed from BARI Gom 28 (BW 28), BARI Chola 5 (BC 5), BARI Mung 6 (BM 6) samples was essential to conduct a fruitful experiment. Keeping that in mind a few of the physiological parameters such as: characterization (differentiating wastes and pure seed), measuring thousand-grain weight, determination of moisture and dry matter content of grains are done. Obtained results are shown in Table 1.

**Table 1:** Physiological parameters of food grains.

Seed type	Characterization of seed		Thousand grain weight	Moisture content (MC) and dry matter content (DM) of the seed	
	Pure seed (%)	Wastage (%)	Weight (g)	MC (%)	DM (%)
BW 28	94.99 ± 1.05	5.12 ± 0.11	50.63 ± 0.07	11.11 ± 0.29	89.16 ± 0.30
BC 5	83.25 ± 0.15	17.23 ± 0.41	128.84 ± 0.05	12.5 ± 0.09	87.60 ± 0.12
BM 6	97.32 ± 0.57	3.02 ± 0.07	56.51 ± 0.04	9.49 ± 0.06	90.38 ± 0.23

In case of characterizing, 94.99 ± 1.05% pure and 5.12 ± 0.11% wastage are found for BW 28 grains. Similarly, 83.25 ± 0.15%purity and 17.23 ± 0.41% wastage are found for BC 5 and 97.32 ± 0.57%purity and 3.02 ± 0.07% wastage are found for BM 6 grains. In case of thousand grain weight, BW 28, BC 5 and BM 6 grains showed 50.63 ± 0.07g, 128.84 ± 0.05g and 56.51 ± 0.04g weight respectively. Finally, BW 28, BC 5 and BM 6 grains showed 11.11 ± 0.29%, 12.5 ± 0.09% and 9.49 ± 0.06% MC, and 89.16 ± 0.30%, 87.60 ± 0.12% and 90.38 ± 0.23% DM content respectively.

## Germination assay

### Germination percentage (GP) and sprout length (SL)

**Figure 1:**
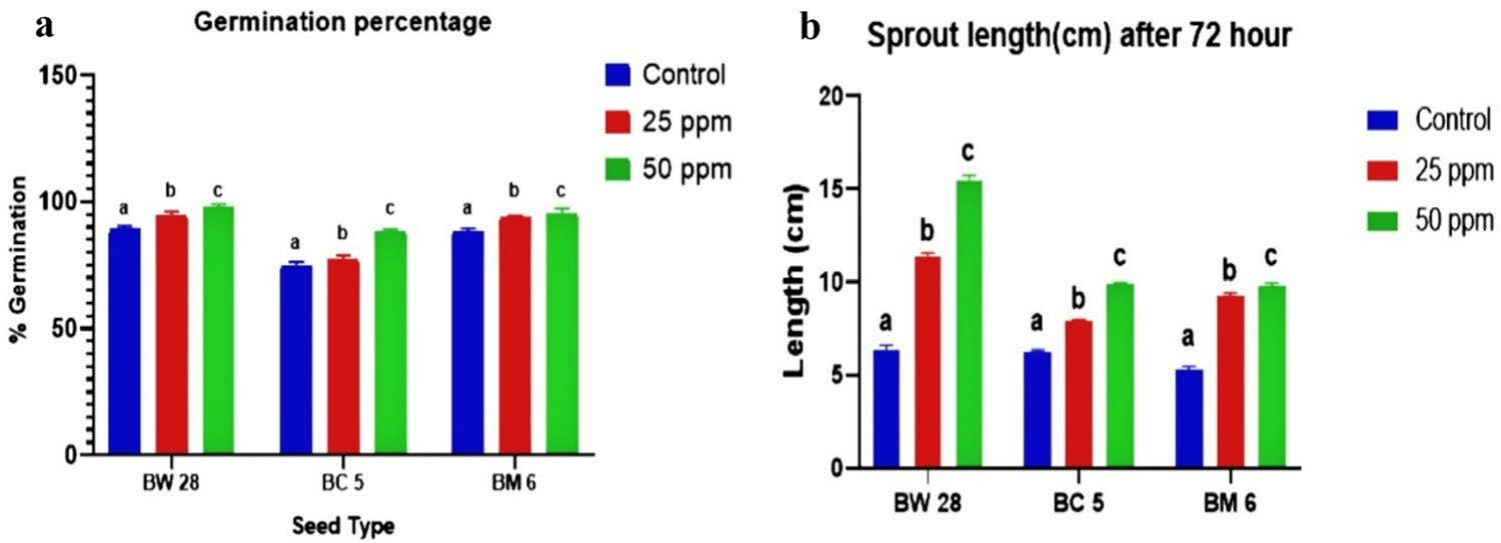
(**a**) Germination percentage of BW 28, BC 5 and BM 6 after 72h of germination under control, 25 ppm and 50 ppm Zn treatment with mean ± SD (*p* ≤ 0.05) (**b**) Sprout length of BW 28, BC 5 and BM 6 sprouts under control, 25 ppm and 50 ppm Zn treatment at 72h period with mean ± SD (*p* ≤ 0.05).

After successful germination of 72h period, BW 28, BC 5 and BM 6 showed minimum GP of 89.15 ± 1.38%, 74.76 ± 1.34%, and 88.36 ± 1.02% at control and maximum GP of 97.68 ± 1.43%, 87.87 ± 1.31%and 95.37 ± 1.93% at 50 ppm Zn treatment respectively (Fig. 1a).Sprout length of each variety was measured after 72h treatment. BW 28, BC 5 and BM 6 showed minimum SL of 7.23 ± 0.34 cm, 6.93 ± 0.23 cm, 5.29 ± 0.18 cm at control and maximum SL of15.45 ± 0.29 cm, 9.59 ± 0.34 cm and 9.44 ± 0.29 cm at 50 ppm Zn treatment respectively (Fig. 1b).

Application of Zn treatment showed significant effect on GP and SL after 72h of germination, all sprouts of BW 28, BC 5 and BM 6 grains with 50 ppm Zn treatment showed the highest reading and as the concentration of Zn treatment increased, the GP and SL increased accordingly. Useful datasets for GP and SL are presented in supplementary Table S1 and Table S2 respectively.

### Fresh and dried weight of sprout

**Figure 2:**
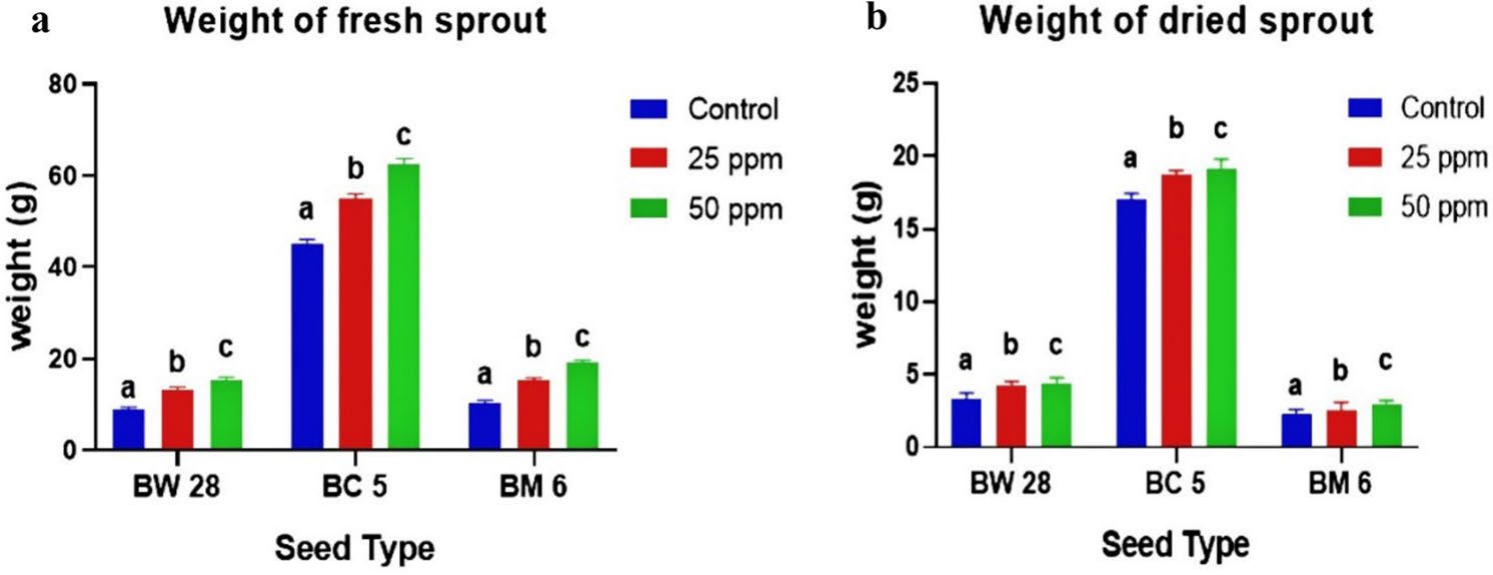
(**a**) Weight of 100 fresh sprouts of BW 28, BC 5 and BM 6 under control, 25 ppm and 50 ppm Zn treatment with mean ± SD (*p* ≤ 0.05) (**b**) Weight of 100 dried sprouts of BW 28, BC 5 and BM 6 under control, 25 ppm and 50 ppm Zn treatment with mean ± SD (*p* ≤ 0.05).

One hundred fresh BW 28, BC 5 and BM 6 sprouts showed minimum fresh weight (FW) at control treatment which are 9.1 ± 0.1g, 45.1 ± 0.2g and 10.1 ± 0.1g and maximum FW at 50 ppm Zn treatment which are 15 ± 0.1g, 62.03 ± 0.15g and 19 ± 0.1g respectively after 72h of germination. Similarly, one hundred dried sprouts of BW 28, BC 5 and BM 6 sprouts showed minimum dried weight (DW) at control treatment which are 3.73g ± 0.09g, 17.08 ± 0.07g and 2.08 ± 0.08g, and maximum DW at 50 ppm Zn treatment which are 4.37 ± 0.04g, 19.05 ± 0.05g and 3.02 ± 0.05g respectively (Fig. 2a,b).

Application of Zn has shown a positive effect on FW and DW. Sprouts of BW 28, BC 5 and BM 6 sprouts with 50 ppm Zn treatment showed the highest reading and as the concentration of Zn treatment increased, the FW and DW progressed accordingly. Among grain varieties, BC 5 has shown the highest FW and DW values. Useful datasets for the weight of fresh and dried sprouts are presented in supplementary Table S3 and Table S4 respectively.

### Vigor index (VI) and biological yield (BY)

**Figure 3:**
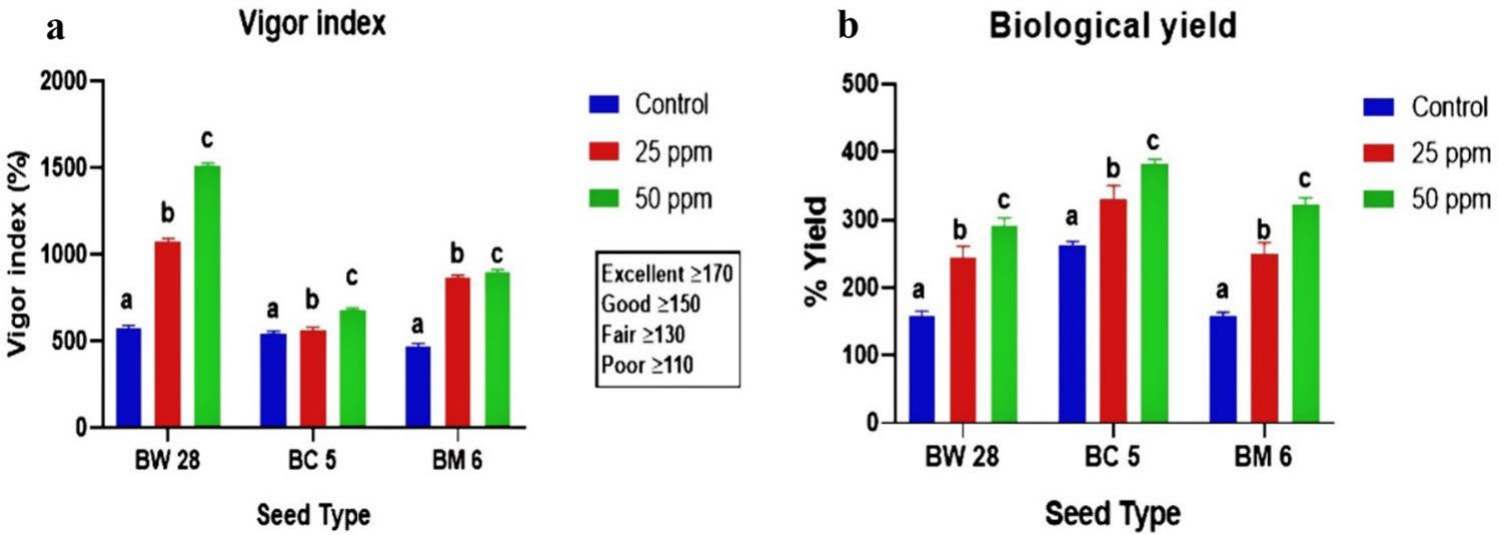
(**a**) Vigor index of BW 28, BC 5 and BM 6 sprouts under control, 25 ppm and 50 ppm Zn treatment with mean ± SD (*p* ≤ 0.05) (**b**) Biological yield of BW 28, BC 5 and BM 6 sprouts under control, 25 ppm and 50 ppm Zn treatment with mean ± SD (*p* ≤ 0.05).

BW 28 sprouts showed minimum VI (570.14 ± 22.85) at control and maximum VI (1510.08 ± 15.03) at 50 ppm Zn treatment. BC 5 sprouts showed minimum VI (545.77 ± 14) at control and maximum VI (678.54 ± 13.67) at 50 ppm Zn. BM 6 sprouts showed minimum VI (473.36 ± 15.22) at the control and maximum VI (897.76 ± 14.55) at 50 ppm Zn treatment (Fig. 3a). Comment on the seed vigority was interpreted according to a previous study^16^. On the other hand, BW 28 sprouts showed minimum BY (159.04 ± 6.86%) at control and maximum BY (290.70 ± 13.75%) at 50 ppm Zn treatment. BC 5 sprouts showed minimum BY (263.84 ± 4.22%) at control and maximum BY (382.44 ± 7.05%) at 50 ppm Zn. BM 6 sprouts showed minimum BY (157.85 ± 5.78%) at control and maximum BY (323.35 ± 9.64%) at 50 ppm Zn treatment (Fig. 3b).

Zn treatment has a significant effect on seed VI and BY. BW 28, BC 5 and BM 6 grains showed “Excellent” level of seed vigority. Progression of Zn treatment level and response of VI and BY showed a positive correlation. Useful datasets for VI and BY are presented in supplementary Table S5 and Table S6 respectively.

### Determination of Zn concentration

In the edible portion (seed), Zn concentration is found to be maximum (36 ppm) at 50 ppm Zn and minimum (0.56 ppm) at control treatment for BW 28 samples. BC 5 samples showed maximum Zn concentration (44.50 ppm) at 50 ppm Zn and minimum (0.57 ppm) at control treatment. BM 6 samples showed maximum Zn concentration (39.50 ppm) at 50 ppm Zn and minimum (0.72 ppm) at control treatment.Similarly, in the inedible portion (root), Zn concentration is found to be maximum (33 ppm) at 50 ppm Zn and minimum (0.53 ppm) at control treatment for BW 28 samples. BC 5 samples showed maximum Zn concentration (41.50 ppm) at 50 ppm Zn and minimum (0.52 ppm) at control treatment. BM 6 samples showed maximum Zn concentration (33.50 ppm) at 50 ppm Zn and minimum (0.45 ppm) at control treatment. On the whole, maximum Zn concentration is found in edible seed portion of BC 5 samples under 50 ppm Zn and minimum Zn is found in inedible root portion of BM 6 samples under control treatment (Table 2).

**Table 2:** Extracted Zn concentrations of sprouts.

Sprout sample	Treatments		
	Control (0 ppm)	25 ppm	50 ppm
BARI Gom 28 (BW 28)			
Edible portion (Seed)	0.56	17	36
Inedible portion (Root)	0.53	15.50	33
BARI Chola 5 (BC 5)			
Edible portion (Seed)	0.57	22.50	44.50
Inedible portion (Root)	0.52	20.50	41.50
BARI Mung 6 (BM 6)			
Edible portion (Seed)	0.72	19.50	39.50
Inedible portion (Root)	0.45	16	33.50

Numerical results clarified that, biofortifying of BW 28, BC 5 and BM 6 grains in this experiment is successful. Certain amount of Zn is found in both edible seed and inedible root portion of all grain sprouts and level of this extracted Zn rises with gradually increasing of Zn treatment.

### Determination of Zn bioaccessibility

BW 28 seed samples showed highest (72%) at 50 ppm Zn and root samples showed lowest (62%) bioaccessibility of Zn at 25 ppm Zn treatment. BC 5 seed samples showed maximum (90%) and root samples showed minimum (82%) Zn bioaccessibility at 25 ppm Zn treatment. Similarly, BM 6 seed samples showed maximum (79%) at 50 ppm and root samples showed minimum (64%) Zn bioaccessibility at 25 ppm Zn treatment. In view of this, all the seed samples showed higher and root samples showed a lower percentage of Zn bioaccessibility and among all samples, BC 5 seed samples showed the highest and BW 28 root samples showed the lowest percentage of Zn bioaccessibility. However, the bioaccessibility percentage for the control-treated samples was not calculated because the amount of artificially used Zn as treatment here is zero (Table 3).

**Table 3:** Bioaccessibility of Zn in sprouts.

Sprout sample	Treatments					
	Control (0 ppm)		25 ppm		50 ppm	
	Extracted Zn (ppm)	Bioaccessibilit*y* (%)	Extracted Zn (ppm)	Bioaccessibilit*y* (%)	Extracted Zn (ppm)	Bioaccessibilit*y* (%)
BARI Gom 28 (BW 28)						
Seed sample	0.56	Not detectable	17	68	36	72
Root sample	0.53	Not detectable	15.50	62	33	66
BARI Chola 5 (BC 5)						
Seed sample	0.57	Not detectable	22.50	90	44.50	89
Root sample	0.52	Not detectable	20.50	82	41.50	83
BARI Mung 6 (BM 6)						
Seed sample	0.72	Not detectable	19.50	78	39.50	79
Root sample	0.45	Not detectable	16	64	33.50	67

It is clearly understood from the obtained results is that, significant amount of bioaccessible percentage of Zn is assessed after successful biofortification, wet digestion and atomic absorption spectrophotometer analysis of BW 28, BC 5 and BM 6 sprout. Progression of bioaccessible percentage of Zn with the increasing of Zn treatment level showed a positive correlation.

### Statistical optimization of Zinc bioaccessibility

Statistical analysis was done to optimize and identify which factor has a significant effect, and which plant part of which variety will give the highest response to Zn at which treatment. In this analysis, the R squared, R squared (adjusted) and R squared (predicted) are 97.90%, 95.91% and 91.58%respectively at 95% confidence level (Table 4). The results show that there are significant effects of both variety and treatment on bioaccessibility of Zn. Useful dataset for statistical analysis (experimental design for fractional factorial design analysis with two replications) is presented in supplementary Table S7.

**Table 4:** Model summary and analysis of variance (ANOVA).

Model summary					
S	R-sq	R-sq(adj)	R-sq(pred)		
3.39400	97.90%	95.91%	91.58%		
Analysis of variance (ANOVA)					
Source	DF	Adj SS	Adj MS	*F*-Value	*P*-Value
Model	17	9647.58	567.50	49.27	0.000
Linear	5	9530.07	1906.01	165.46	0.000
Variety	2	76.36	38.18	3.31	0.059
Plant part	1	13.24	13.24	1.15	0.298
Zn treatment	2	9440.47	4720.24	409.77	0.000
2-Way Interactions	8	85.29	10.66	0.93	0.519
Variety*Plant part	2	24.01	12.01	1.04	0.373
Variety*Zn treatment	4	55.46	13.87	1.20	0.343
Plant part*Zn treatment	2	5.82	2.91	0.25	0.780
3-Way Interactions	4	32.21	8.05	0.70	0.603
Variety*Plant part*Zn treatment	4	32.21	8.05	0.70	0.603
Error	18	207.35	11.52		
Total	35	9854.93			

### Screening of factors

**Figure 4:**
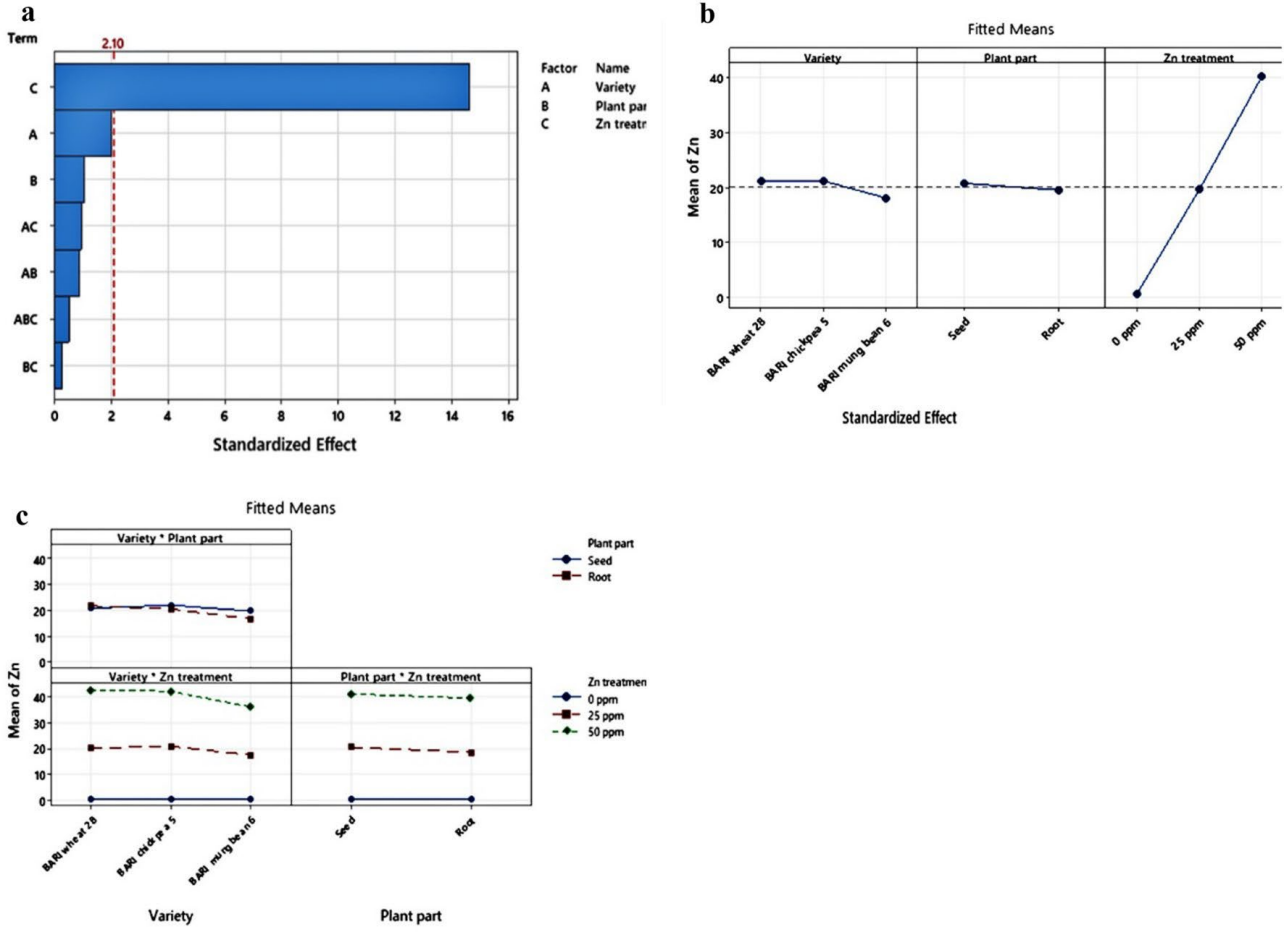
(**a**) Pareto chart of the standardized effects. (**b**) Main effects plot for Zn. (**c**) Interaction plot for Zn.

Significance level of the effects of three factors (Zn treatment, plant part and variety) on the physiological parameters and maximum Zn response of food grains is shown by the Pareto chart (Fig. 4a). The factor “Zn treatment” has the highest level of significant effect, the factor “variety” has comparatively lower effect and the factor “plant part” has the lowest level of significant effect on the physiological parameters and maximum Zn response. On the other hand, the effect of other interactions between these three factors is insignificant. The main effects plot for Zn and the interaction plot for Zn provide us that BC 5 among the varieties, seed between the plant parts and 50 ppm Zn among Zn treatments have higher Zn response (Fig. 4b,c).

### Optimization of multi-level fractional factorial design analysis

**Figure 5:**
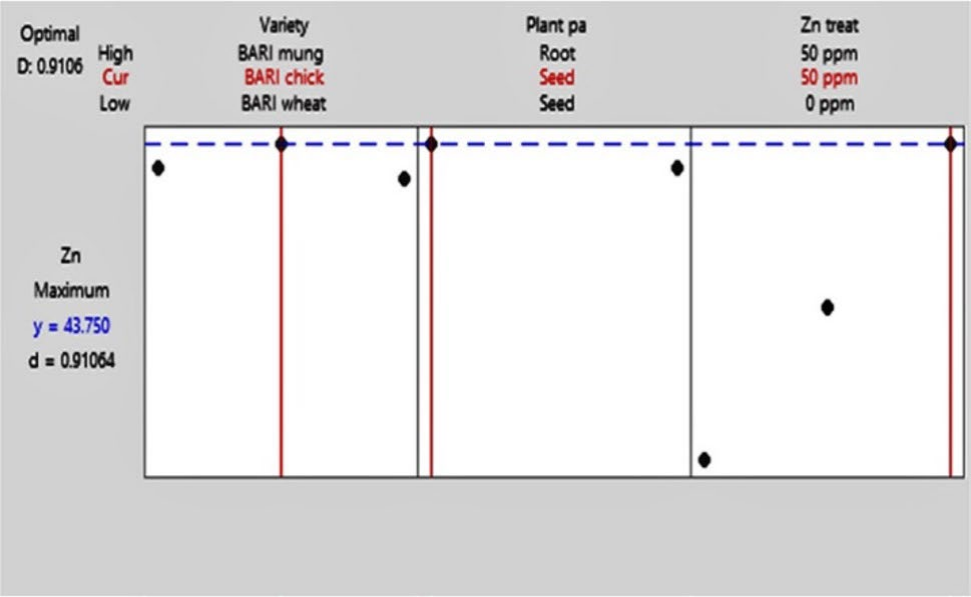
Optimization plot for fractional factorial design analysis.

Screening of the factors with two replications provided us with 36 groups of data from 18 samples which were then optimized to find out the best result among them. The maximum response for Zn has observed in the seed of BC 5 at 50 ppm Zn treatment among BW 28 and BC 5 varieties, between seed and root plant parts and among control, 25 ppm and 50 ppm Zn treatments. The composite desirability in this analysis is 91.06% (Table 5 and Fig. 5).

**Table 5:** Optimization of multi-level fractional factorial design analysis.

Parameters										
Response		Goal	Lower		Target		Upper	Weight		Importance
Zn		Maximum	0.44		48			1		1
Variable ranges										
Variable			Values							
Variety			BARI Gom 28, BARI Chola 5							
Plant part			Seed							
Zn treatment			0 ppm, 25 ppm, 50 ppm							
Solution										
Solution	Variety			Plant part		Zn treatment		Zn Fit	Composite desirability	
1	BARI Chola 5			Seed		50 ppm		43.75	0.910639

## Discussion

Current study presents an approach toprofile the level and bioaccessible percentage of Zn in the edible (seed) and inedible (root) portion of the germinated sprouts after biofortification and to assess a few germination assays of BW 28, BC 5 and BM 6 food grains by hydroponics approach using control, 25 ppm and 50 ppm Zn treatments.

In case of physiological assay of BW 28, BC 5 and BM 6 grains, it was reported that 91% purity in a seed lot means that the seed is of best quality^17^. As per the reported result, it can be said that the supplied seed lot used for current study is of best quality. However, slight deviation may have happened due to storage and environmental differences. Thousand-grain weight content of food grain is one of the most important scales in seed quality that allows us to understand about germination percent, seedling emergence and yield production^18^. In a previous study it was reported that, thousand grain weight of BW 28, BC 5 and BM 6 should be 43-48g, 110-120g and 51-52g respectively, which are similar to the experimental findings^19–21^. In accordance with this previous report, BW 28, BC 5 and BM 6 seeds are considered to be of best quality with a good emergence and production rate. It was also reported in a few prior studies that MC of BW 28, BC 5 and BM 6 should be 10–12%, 11%, 8.7%^22–24^ and DM should be 83%, 85% and 92% respectively^19–21^. These previously reported results corroborate with our experimental results.

In view of germination percentage (GP) and sprout length (SL), it is reported that wheat of different varieties usually have 76–98% germination^25^ and also usual GP of chickpea is 70–95%, mung bean is 87–96% which supports our experimental findings^26,27^. It has also been reported in previous studies that usual SL of wheat, chickpea and mung bean is 1.07–3.71 cm, 8.50–9.82 cm and 3.33–8.85 cm respectively, which shows the slight difference from our experimental findings that may have been caused due to dissimilarity of experimental design and variety^28–30^.

Fresh weight (FW) of wheat sprouts is reported to be in between 21.86–54.80g after 7days of germination and dried weight (DW) is 2.13–5.93g in a previous study^31^. FW of 500g chickpea sprouts is 1119g and DW is 7.18–11.25g^30,32^ and FW of mung bean sprouts is 23.80–46.68g after 4 months of germination and DW is 2.6g^33^. Suggested DWs support our experimental value whereas FWs may differ due to differences in experimental design.

In case of vigor index (VI) and biological yield (BY), it is reported that VI of wheat is 1501.20–1659, mung bean is 1437 and chickpea is 550^28,32,34^. Suggested values correlate with our experimental values with slight differences due to deviation in variety. It is also reported that the BY of wheat is 523.8–561.7 under plasma treatment, mung bean/plant is 0.518–0.670 in the rainy season and chickpea is 2.1–3 t/ha^27,32,34^. Suggested values may differ due to differences in plant variety, environmental conditions and experimental design.

Naturally or control-treated wheat usually contains 1.73–53.05 ppm Zn and Zn biofortified sprouts contain 33.1–63.1 ppm Zn^35^. It is also reported that, naturally chickpea contains 1.5 ppm Zn and when it is treated with Zn the edible portion (seed) of chickpea sprouts contains 39.05–44.98 ppm Zn and the inedible portion (root) contains 34.61–44.08 ppm Zn^36^. Natural mung bean contains 1.7 ppm Zn and if it goes through Zn biofortification then the Zn content rises up to 17.01–54.91 ppm^37^. Suggested values correlates with our experimental findings and slight deviation may have happened due to difference in variety and experimental design.

In a research article, it is reported that the bioaccessibility percent of Zn in wheat grain is 8.90%, chickpea is 56.50% and mung bean is 41%^38^. The possible reason behind these deviations between experimental value and the suggested value may be due to the difference in experimental design and genetic variation.

In case of statistical analysis, it is reported in a previous article that any scientific experiment that has R-squared value above 90% is good and reliable^39^. This ensures the reliability and acceptability of this experiment and the analysis. Result interpretability and direct correspondence with the stated goals of fractional factorial designs offer a high probability of correctly selecting the important factors.

Optimization of applied simulation permits us to generate designs from one of the proposed criteria. This eventually helps in identifying factors by any one of several analysis procedures that would correspond to correct selection of assumptions about the studied system and selected analysis method^40^. This states the authenticity and correct selection of assumption about which plant part between seed and root of which variety among BW 28, BC 5 and BM 6 will receive the highest amount of Zn for better growth, yield and many other physiological abilities from which Zn treatment among control, 25 ppm and 50 ppm Zn.

The results of the present study reveals that hydroponics approach for biofortifying BW 28, BC 5 and BM 6 grains and developing sprout is an effective Zn biofortification method. ZnO at 50 ppm concentration was the best Zn treatment comparing to 25 ppm and lastly control treatment because both of the edible (seed) and inedible (root) part of treated sprouts showed the highest amount of Zn content and Zn bioaccessibility in them under this treatment. Furthermore, several observed germination assays (GP, SL, FW, DW, VI and BY) also showed the best results under 50 ppm Zn followed by 25 ppm Zn and lowest results at control treatment. These results suggest that expected better outcome for Zn concentration, Zn bioaccessibility, other physical and physiological development of BW 28, BC 5 and BM 6 grains is best feasible at 50 ppm Zn followed by 25 ppm Zn treatment and control treatment did not give better outcome.

## Conclusion

In Bangladesh, the national status of Zn deficiency is severe. 44.6% of preschool children, 57.3% of adult men and 66.4% of adult women suffer from Zn deficiency which is both alarming and dangerous^41^ and mass people of Bangladesh cannot get enough Zn rich animal food because of the low income issue. Keeping the above in mind, current investigation is undertaken to develop Zn biofortified grain and low cost vegetative source of food which can play an important role in eradicating Zn deficiency from the people of all walks of life in Bangladesh. Effect of Zn treatments (Control, 25 ppm and 50 ppm Zn) is evaluated in the germination assay (germination percentage, biological yield, sprout length, fresh and dried weight of sprout, seed vigority) of BW 28, BC 5 and BM 6 food grains. After determination of Zn concentration, bioaccessible Zn percentage calculation and statistical analysis it become clear that the seed of BC 5 sprouts under 50 ppm Zn treatment showed maximum Zn response. Further studies will be essential to formulate an easily accessible human food (bread, cake, pasta, noodles, and biscuit), infant food supplement, oral saline, etc. using this Zn biofortified BC 5 flour to eradicate the Zn deficiency of mass people of Bangladesh considering availability and all the biosafety issues along the processes.

## Materials and methods

### Sample and chemicals

Wheat (BARI Gom 28), chickpea (BARI Chola 5) and mung bean seeds (BARI Mung 6) were collected from Bangladesh Agricultural Research Institute (BARI), Joydebpur, Gazipur. These plant materials are the breeds improved by BARI, widely cultivated throughout Bangladesh and are not endangered species. These plant materials have been collected through proper channel that complies with all relevant institutional, national, IUCN and other international guidelines and legislation.

All experiments excluding the wet digestion, AAS analysis and bioaccessibility determination of Zn were carried out in the Food Chemistry and Analysis Laboratory at the Department of Food Technology and Rural Industries in Bangladesh Agricultural University. Wet digestion of sprouts was carried out in the laboratory of the Soil Resource Development Institute (SRDI), Mymensingh. AAS analysis and bioaccessibility determination of Zn in the biofortified sprouts of food grain were done in the Postgraduate Laboratory-2 at the Department of Agricultural Chemistry in Bangladesh Agricultural University. Nitric acid (HNO_3_), Per Chloric Acid (HClO_4_), Zn oxide (ZnO), sodium hypochlorite (NaOCl) and other chemicals used in this study were of laboratory grade and from Merck, India and Shell, England.

### Preparation of zinc oxide (ZnO) solution

Three different Zn treatments, i.e., control, 25 ppm and 50 ppm Zn were used in this study and were prepared with modifications to a previous study^42^. Two ZnO solutions of different concentrations were freshly prepared by dispersing 25mg and 50mg of ZnO oxide nanoparticles into 1 L of deionized water for 25 ppm and 50 ppm Zn solutions respectively. After that, the particles in deionized water were treated by ultrasonic vibration (100 w, 40 kHz) for 30 min usingpowersonic610. Modification was applied in repeating the ultra-sonication treatment for two times. Zn concentrations used in this study were prepared according to Raskar et al*.*^43^.

### Upgradation of food grains

Selected food grains were characterized and unwanted materials like; soil adhering to seed coat, mixed varieties, diseased seeds, weeds, dirt and dead insects, etc. were opted out as per a previous study^44^.One thousand uniform and characterized pure seeds were counted and weighed accurately by using a digital electronic balance with three replications. Thousand grain weight of BARI Gom 28 (BW 28), BARI Chola 5 (BC 5) and BARI Mung 6 (BM 6) seeds have been determined according to a previously reported method^18^.

### Seed surface sterilization

Before germination, the seed surface was sterilized to remove the minute presence of *Aspergillus flavus* or *Aspergillus parasiticus*, which are responsible for causing the aflatoxicity in seeds. As per Rai-Kalal and Jajoo^42^,seeds were sterilized by using a 10% sodium hypochlorite (NaOCl) solution for 3 min and then carefully washed with deionized water to remove all the chloride contents. To ensure the complete removal of chloride content from the seed surface, this washing was done in triplicate.

### Dry matter and moisture content determination

The dry matter and moisture content of BW 28, BC 5 and BM 6 seeds were determined according to a previous method with a few modifications^45^. 2 g of seed sample was taken from each variety. They were oven-dried at 130 °C for 6 h in total (2 h for each grain variety). Prior to that, the weight of the empty container (W_1_), and the weight of the container with grains in it before drying (W_2_) were taken. After drying, the weight of the container with grains in it (W_3_) was taken too. Finally, the moisture content of the seed sample was calculated by formula (1) and the dry matter content of the seeds was then calculated according to formula (2)

1 $$\% \,Moisture \,content = \frac{W2 - W3}{{W2 - W1}} \times 100$$

where.

*W*_2_–*W*_3_ = Moisture loss.

*W*_2_–*W*_1_ = Fresh weight of sample.

*W*_1_ = Weight of the empty container.

*W*_2_ = Weight of the container with the seed materials in it before drying.

*W*_3_ = Weight of the container with the seed materials in it after drying

2 $$\% \,Dry \,matter \,content = 100 - \% \,moisture \,content$$

### Seed viability test

**Figure 6:**
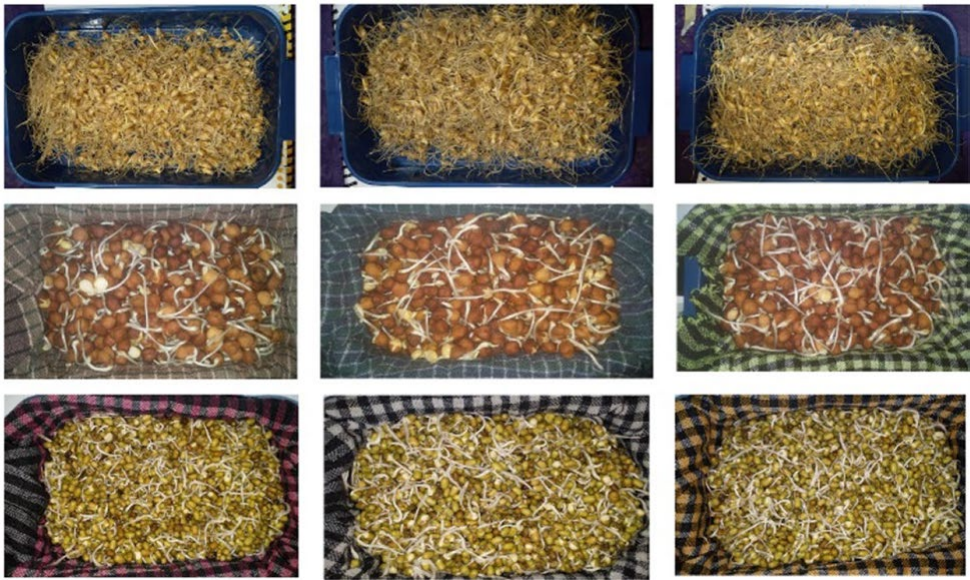
Freshly grown BW 28 sprouts at (**a**) control, (**b**) 25 ppm and (**c**) 50 ppm Zn treatment; BC 5 sprouts at (**d**) control, (**e**) 25 ppm and (**f**) 50 ppm Zn treatment and BM 6 sprouts at (**g**) control, (**h**) 25 ppm and (**i**) 50 ppm Zn treatment. (Left to right side and descending order).

To predict the chance of a successful experiment for the identical BW 28, BC 5 and BM 6 grain, seed viability test is done prior to biofortification through hydroponics approach as per Xu et al.^46^.Firstly, the seeds were selected, rinsed in deionized water and drained. They were then placed in distilled water and soaked overnight in the dark for 8h at 25 ± 3 °C. Modification was applied after pouring off the soaking water. The soaked seeds were rinsed in water for about 5 min and spread evenly on a wet cotton cloth inside a box. Necessary temperature (24–25 °C) and 90–95% relative humidity were maintained by covering them with wet cotton cloth. After 24 h, almost all seeds were germinated and sprouted (Fig. 6). Germination percentage of the seed lot after 72 h of germination was calculated by a formula (3)according to Ujjainkar and Marawar^47^.

3 $$Germination \,percentage = \frac{Germinated \,seeds }{{Total \,no. \,of \,seeds}} \times 100$$

### Fresh and dried weight of sprouts

After 72 h of germination, the weight of fresh BW 28, BC 5 and BM 6 sprouts was measured as per Hui et al*.*^34^ with modifications. 100 fresh and qualitative sprouts of all varieties were measured precisely in triplicate. Dried weight of 100 sprouts was determined according to Al-salhy and Rasheed^28^ with a few modifications too. The sprouts were placed in a hot air oven and dried for 3 days at 65–68 °C temperature. After proper drying, the weight of these 100 sprouts was measured in triplicate accurately. Both of the measurement was taken by using a digital electronic balance. In both case, modifications were applied in obtaining the number of sprouts taken for the measurement.

### Measuring sprout length

**Figure 7:**
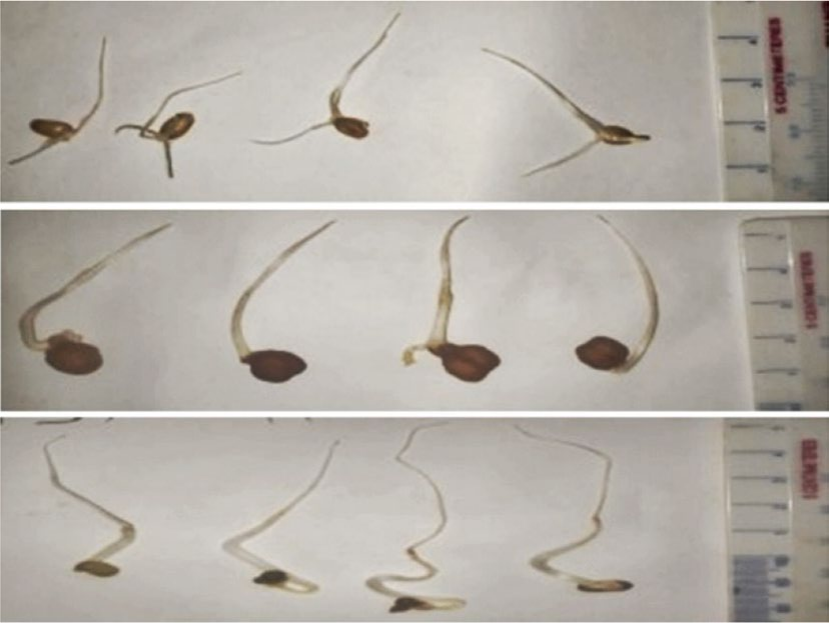
Measuring sprout length of (**a**) BW 28 sprout, (**b**) BC 5 sprout and (**c**) BM 6 sprout after 72h of germination using “Image J”. (Descending order).

Root length of the sprouted seeds was measured according to Kimura et al.^48^ with modifications. An image analysis system, consisting of a personal computer (Hewlett Packard with Intel core i3 7th generation 2.7 GHz processor) and a public domain program NIH Image 1.61 (Image J) developed by the U.S. National Institutes of Health. Images were captured by12-megapixel camera using an android device. For image acquisition, roots were placed on white paper with a scale showing centimeter lengths on the right side of the frame. Finally, by using the “Image J” software, root lengths of the sprout samples of each treatment were measured after 24 h, 48 h and 72 h (Fig. 7).

### Seed vigor index

After 3 days of germination, the sprout length and germination percentage were taken into account for determining the seed vigor index. Then seed vigor index was calculated using Eq. (4) from Kataria et al*.*^49^ and seed vigority was determined according to a seed vigor index as per Norman hopper^16^.

4 $$Vigor \,index \,1 = Percent \,germination \times Sprout \,length$$

### Biological yield

The biological yield for each variety of seed for three different treatments was calculated using Eq. (5) according to Zou et al*.*^50^.

5 $$Biological \,yield \,\% = \frac{FW}{M} \times 100$$

Here

*FW* = Fresh weight of sprouts.

*M* = Weight of seeds used for germination in each trial.

### Biofortification of food grains

Biofortification or priming of seed through hydroponics approach was done as per Zou et al*.*^50^ with modifications. Firstly, identical seed were rinsed in deionized water and drained. Then seed of each variety was separated into three groups for their treatment by control, 25 ppm and 50 ppm Zn solution. After that BW 28, BC 5 and BM 6 seed were soaked overnight in targeted priming media in dark for 8h at 25 ± 3 °C. After pouring off the soaking solution, soaked seeds were rinsed in running water (25 ± 3 °C) for about 5 min and spread evenly on a wet cotton cloth inside a box. Prior to that, all nine cotton cloths were sterilized and soaked with priming media. Then to maintain the necessary temperature (24–25 °C) and 90–95% relative humidity, the soaked seeds were covered with wet cotton cloths. After 24 h of this treatment, almost all seeds started germinating and the biofortification continued for all three varieties of seed with targeted priming media. After 72 h of biofortification, treated sprouts were directed to the next phase of this experiment.

### Zinc profiling and bioaccessibility

The appropriate method for the determination of mineral constituents in plant extract is the wet oxidation approach using di-acid mixture of nitric and per-chloric acid. Nitric acid supplies greater part of the required amount of oxygen for oxidation which softens the plant tissue and per-chloric acid aids in digestion because it breaks down a few of the organic compounds into simple ones which more readily become oxidized by nitric acid^51^.

### Sample preparation

Sample preparation to profile the amount of zinc present in the inedible (root) part and edible (seed) part of the wheat, chickpea and mung bean sprouts and production of di-acid mixtures by using nitric acid and perchloric acid in a mixture of 2:1 ratio (HNO_3_:HClO_4_ = 2:1) were done according to Paul^51^. Freshly sprouted seeds were dried at 62–64 °C for 72 h in a cabinet dryer. Then the dried sprouts of BW 28, BC 5 and BM 6 of three different treatments (Control, 25 ppm and 50 ppm Zn) were divided into 18 groups having the root and seed part of the sprouts being separated. After that, they were powdered to a fine grade using a stainless-steel grinder and passed through a 1mm sieve.

### Complete digestion of sample

The digestion approach applied in this study was completed as per Paul^51^ with modification. 1g of previously prepared dried plant sample is taken into a 250ml conical flask. 30ml of the di-acid mixture (HNO_3_:HClO_4_ = 2:1) is added into the conical flask and the flask is stirred to moisten the plant part in it. Then the flask is placed on an electric sand bath and heated at 180–200 °C until the white fumes appeared. Then the heated analyte is kept at 250 °C on an electric hot plate till the solution became translucent. About 5ml of the di-acid mixture is added to the flask as the content almost became dried before the end of digestion. After that, the flask is removed from the hot plate and allowed to cool. Then 20ml of distilled water is added to the conical flask and shaken thoroughly. The solution is then filtered using filter paper (Whatman no. 1) into a 50ml volumetric flask afterwards. The conical flask is washed three times to ensure the transfer of all minerals into the volumetric flask. Finally, the volume is made up to the mark with distilled water. Thus, the digestion process become completed and the sample is prepared for the determination of bioaccessibility and profiling of Zn.

### Determination of Zn content by AAS

The presence of Zn content in freshly prepared 18samples of BW 28, BC 5 and BM 6 sprouts are determined by atomic absorption spectrophotometer (AAS) analysis. Cautions in sample preparation and sample handling during filtration were followed according to Isaac and Kerber^52^. 50ml solutions of all samples were analyzed in triplicate by an AAS naming Shimadzu, AA-7000 with an auto-sampler system, Shimadzu, ASC-7000.

### Determination of Zn bioaccessibility percentage

Followed by the determination of Zn content by atomic absorption spectrophotometer (AAS), the bioaccessible percentage of Zn in BW 28, BC 5 and BM 6 were calculated using Eq. (6) according to Zou et al*.*^50^.

6 $$Bioaccessibility \,percentage = \frac{Zinc \,content \,in \,dialysate}{{Total \,zinc \,concentration \,applied}} \times 100$$

### Statistical analysis

Statistical analysis of data obtained after AAS analysis and bioaccessibility percentage calculation in two replication provides us with 36 groups of data from 18 samples of BW 28, BC 5 and BM 6 sprouts. This analysis was done by fractional factorial design analysis approach as per Jiju antony^53^ at 95% confidence level. The combination of factors from fractional factorial design and their optimization was analyzed by using Minitab Statistical Software Version 21.1.0, data analysis for germination assays was done using IBM SPSS Statistics 20 and graphical representation for better visualizing the effect of Zn treatment on germination assays was done using GraphPad Prism 8.

### Optimization of fractional factorial design analysis

The optimization of fractional factorial design analysis data sets is done according to Antony^54^ with modifications. The three varieties, plant part and Zn treatment were taken as the factors with 3 levels (BW 28, BC 5 and BM 6), 2 levels (seed and root) and 3 levels (control, 25 ppm and 50 ppm Zn) respectively. Modification is applied in specifying the levels of pre-determined factors.

## Supplementary Information

Supplementary Tables.

## Data Availability

The complete data sets generated and analyzed during this current investigation that support the findings of this study are provided in the supplementary information of this article.
