# Supplementary material for: Assessment of the bioaccessibility of zinc in the selected biofortified food grains
Source: Sci Rep. 2025 Mar 3;15:7457. doi: 10.1038/s41598-024-67856-3 (PMC11876695; doi:10.1038/s41598-024-67856-3)
Supplement: Supplementary file 1 — Supplementary Tables. [file 41598_2024_67856_MOESM1_ESM.pdf]

ASSESSMENT OF THE BIOACCESSIBILITY OF ZINC IN THE SELECTED BIOFORTIFIED FOOD GRAINS

by

Kazi NS Rafi[1*], Aziz MG[1], Mohammad Amirul Islam[2], Sarif Istiak Akash[1], Md. Jakariya[3], Moupia Rahman[3]

1. Department of Food Technology and Rural Industries, Bangladesh Agricultural University, Mymensingh-2202, Bangladesh.
2. Department of Agricultural and Applied Statistics, Bangladesh Agricultural University, Mymensingh-2202, Bangladesh
3. Department of Environmental Science and Management, North South University, Dhaka-1229, Bangladesh.

*E-mail: kazi43656@bau.edu.bd, ORCID Id: 0009-0009-1305-1935

Supplementary data sets

Necessary information,

BARI Gom 28 = *Triticum aestivum* = BW 28

BARI Chola 5 = *Cicer arietinum* = BC 5

BARI Mung 6 = *Vigna radiata* = BM6

Supplementary Table S1: Data set for germination percentage (Graphical representation (**Fig- 1a**) in manuscript).

Grain variety	Zn Treatment		
	**Control**	**25 ppm**	**50 ppm**
**BW 28**	89.15±1.38	94.64±1.27	97.68±1.43
**BC 5**	74.76±1.34	77.05±1.70	87.87±1.31
**BM 6**	88.36±1.02	93.69±0.89	95.37±1.93

Supplementary Table S2: Data set for sprout length (Graphical representation (**Fig- 1b**) in manuscript).

Grain variety	Zn Treatment		
	**Control**	**25 ppm**	**50 ppm**
**BW 28**	7.23±0.34	11.39±0.17	15.45±0.29
**BC 5**	6.93±0.23	7.35±0.30	9.59±0.34
**BM 6**	5.29±0.18	9.25±0.16	9.44±0.29

**Supplementary Table S3:** Data set for weight of fresh sprout (Graphical representation (**Fig- 2a**) in manuscript).			
**Grain variety**	**Zn Treatment**		
	**Control**	**25 ppm**	**50 ppm**
**BW 28**	9.1±0.1	13±0.1	15±0.1
**BC 5**	45.1±0.2	55.1±0.1	62.03±0.15
**BM 6**	10.1±0.1	15.1±0.1	19±0.1

**Supplementary Table S4**: Data set for weight of dried sprout (Graphical representation (**Fig- 2b**) in manuscript).			
**Grain variety**	**Zn Treatment**		
	**Control**	**25 ppm**	**50 ppm**
**BW 28**	3.73±0.09	4.28±0.07	4.37±0.04
**BC 5**	17.08±0.07	18.65±0.09	19.05±0.05
**BM 6**	2.08±0.08	2.81±0.07	3.02±0.05

**Supplementary Table S5:** Data set for vigor index (Graphical representation (**Fig- 3a**) in manuscript).			
**Grain variety**	**Zn Treatment**		
	**Control**	**25 ppm**	**50 ppm**
**BW 28**	570.14±22.85	1075.68±20.85	1510.08±15.03
**BC 5**	545.776±14.00	559.53±13.66	678.54±13.67
**BM 6**	473.36±15.22	867.62±15.60	897.76±14.55
**Comment**	All three of these grain varieties have shown “excellent” level of seed vigority.		

**Supplementary Table S6:** Data set for biological yield (Graphical representation (**Fig- 3b**) in manuscript).			
**Grain variety**	**Zn Treatment**		
	**Control**	**25 ppm**	**50 ppm**
**BW 28**	159.04±6.86	244.30±13.75	290.70±13.75
**BC 5**	263.84±4.22	331.91±15.59	382.44±7.05
**BM 6**	157.85±5.78	248.85±13.95	323.35±9.64

Supplementary Table S7: Data set for statistical analysis (Experimental design for fractional factorial design analysis with two replications).

Std. order	Run order	Pt type	Blocks	Variety	Plant part	Zn treatment	Extracted Zn
1	1	1	1	BW 28	Seed	0 ppm	0.53
2	2	1	1	BW 28	Seed	25 ppm	15.50
3	3	1	1	BW 28	Seed	50 ppm	33.00
4	4	1	1	BW 28	Root	0 ppm	0.57
5	5	1	1	BW 28	Root	25 ppm	22.50
6	6	1	1	BW 28	Root	50 ppm	44.50
7	7	1	1	BC 5	Seed	0 ppm	0.52
8	8	1	1	BC 5	Seed	25 ppm	20.50
9	9	1	1	BC 5	Seed	50 ppm	41.50
10	10	1	1	BC 5	Root	0 ppm	0.52
11	11	1	1	BC 5	Root	25 ppm	20.50
12	12	1	1	BC 5	Root	50 ppm	41.50
13	13	1	1	BM 6	Seed	0 ppm	0.72
14	14	1	1	BM 6	Seed	25 ppm	19.50
15	15	1	1	BM 6	Seed	50 ppm	39.50
16	16	1	1	BM 6	Root	0 ppm	0.45
17	17	1	1	BM 6	Root	25 ppm	16.00
18	18	1	1	BM 6	Root	50 ppm	33.50
19	19	1	1	BW 28	Seed	0 ppm	0.56
20	20	1	1	BW 28	Seed	25 ppm	26.50
21	21	1	1	BW 28	Seed	50 ppm	48.00
22	22	1	1	BW 28	Root	0 ppm	0.53
23	23	1	1	BW 28	Root	25 ppm	16.50
24	24	1	1	BW 28	Root	50 ppm	45.00
25	25	1	1	BC 5	Seed	0 ppm	0.56
26	26	1	1	BC 5	Seed	25 ppm	22.25
27	27	1	1	BC 5	Seed	50 ppm	46.00
28	28	1	1	BC 5	Root	0 ppm	0.52
29	29	1	1	BC 5	Root	25 ppm	20.25
30	30	1	1	BC 5	Root	50 ppm	39.00
31	31	1	1	BM 6	Seed	0 ppm	0.72
32	32	1	1	BM 6	Seed	25 ppm	19.00
33	33	1	1	BM 6	Seed	50 ppm	38.00
34	34	1	1	BM 6	Root	0 ppm	0.44
35	35	1	1	BM 6	Root	25 ppm	15.75
36	36	1	1	BM 6	Root	50 ppm	33.00

**Supplementary Table S8:** Data set for statistical analysis (Factors and numeric results of fractional factorial design analysis with two replications)						
**Std. Order**	**Variety**	**Plant part**	**Zn treatment**	**Extracted Zn**	**FITS1**	**RESI1**
1	BW 28	Seed	0 ppm	0.53	0.545	-0.015
2	BW 28	Seed	25 ppm	15.50	21.000	-5.500
3	BW 28	Seed	50 ppm	33.00	40.500	-7.500
4	BW 28	Root	0 ppm	0.57	0.550	0.020
5	BW 28	Root	25 ppm	22.50	19.500	3.000
6	BW 28	Root	50 ppm	44.50	44.750	-0.250
7	BC 5	Seed	0 ppm	0.52	0.540	-0.020
8	BC 5	Seed	25 ppm	20.50	21.375	-0.875
9	BC 5	Seed	50 ppm	41.50	43.750	-2.250
10	BC 5	Root	0 ppm	0.52	0.520	-0.000
11	BC 5	Root	25 ppm	20.50	20.375	0.125
12	BC 5	Root	50 ppm	41.50	40.250	1.250
13	BM 6	Seed	0 ppm	0.72	0.720	0.000
14	BM 6	Seed	25 ppm	19.50	19.250	0.250
15	BM 6	Seed	50 ppm	39.50	38.750	0.750
16	BM 6	Root	0 ppm	0.45	0.445	0.005
17	BM 6	Root	25 ppm	16.00	15.875	0.125
18	BM 6	Root	50 ppm	33.50	33.250	0.250
19	BW 28	Seed	0 ppm	0.56	0.545	0.015
20	BW 28	Seed	25 ppm	26.50	21.000	5.500
21	BW 28	Seed	50 ppm	48.00	40.500	7.500
22	BW 28	Root	0 ppm	0.53	0.550	-0.020
23	BW 28	Root	25 ppm	16.50	19.500	-3.000
24	BW 28	Root	50 ppm	45.00	44.750	0.250
25	BC 5	Seed	0 ppm	0.56	0.540	0.020
26	BC 5	Seed	25 ppm	22.25	21.375	0.875
27	BC 5	Seed	50 ppm	46.00	43.750	2.250
28	BC 5	Root	0 ppm	0.52	0.520	-0.000
29	BC 5	Root	25 ppm	20.25	20.375	-0.125
30	BC 5	Root	50 ppm	39.00	40.250	-1.250
31	BM 6	Seed	0 ppm	0.72	0.720	0.000
32	BM 6	Seed	25 ppm	19.00	19.250	-0.250
33	BM 6	Seed	50 ppm	38.00	38.750	-0.750
34	BM 6	Root	0 ppm	0.44	0.445	-0.005
35	BM 6	Root	25 ppm	15.75	15.875	-0.125
36	BM 6	Root	50 ppm	33.00	33.250	-0.250
